# Supplementary material for: Surrogate modeling of Cellular-Potts agent-based models as a segmentation task using the U-Net neural network architecture
Source: PLoS Comput Biol. 2025 Nov 3;21(11):e1013626. doi: 10.1371/journal.pcbi.1013626 (PMC12611147; doi:10.1371/journal.pcbi.1013626)
Supplement: Attachment — Submitted filename: Response to Reviewers.pdf [file pcbi.1013626.s003.pdf]

**Reviewers' comments:**

Reviewer's Responses to Questions

**Comments to the Authors:**
**Please note here if the review is uploaded as an attachment.**

Reviewer #1: This submission explores how machine learning can be used to significantly accelerate computationally expensive mechanistic models, focusing on the Cellular Potts Model (CPM). Given the CPM's popularity and broad application, the work addresses an important and timely question with potential implications for simulating larger and more complex biological systems.

The manuscript is well-written and easy to follow. The authors clearly describe the benefits of using a machine learning-based surrogate model, including improved computational speed and the ability to reproduce key features of the CPM such as vessel sprouting, branching behavior, and lacunae contraction. They also acknowledge the model's limitations, notably its inability to prevent gradual lacunae growth—an effect not present in the original model.

The figures are generally clear and informative. However, the numbering of figures in the main text does not align with the numbering in the figure captions, which should be corrected for consistency and ease of reference.

We thank the reviewer for drawing attention to this. This was a problem on our end with uploading and naming the files. We have corrected the figure image names and intend for this to be fixed during resubmission.

Main Concerns

My principal concern is that the success of the surrogate model is overstated in several key parts of the paper. While the authors acknowledge in the main text that the surrogate performs well for only one to three prediction steps, they still present the model as broadly useful in ways that are not fully supported by the results. This limitation substantially reduces the model's practical utility, as the performance gain from faster simulations cannot be fully leveraged over such short prediction horizons.
For example, the abstract states:

"The surrogate model was trained to predict 100 computational steps ahead (Monte-Carlo steps, MCS), accelerating simulation evaluations by a factor of 590 times compared to CPM code execution. Over multiple recursive evaluations, our model effectively captures the emergent behaviors demonstrated by the original Cellular-Potts model such as vessel sprouting, extension and anastomosis, and contraction of vascular lacunae."

While technically accurate, this statement omits the critical issue that the model's predictions deteriorate over longer time horizons, particularly in representing lacunae size. For balance, the abstract should briefly mention these limitations.

We thank the reviewer for their insight and have made the following changes:

**In the language of the abstract:**

From: Over multiple recursive evaluations, our model effectively captures the emergent behaviors demonstrated by the original Cellular-Potts model of such as vessel sprouting, extension and anastomosis, and contraction of vascular lacunae.

To: Over short timescales of up to 3 recursive evaluations, or 300 MCS, our model captures the emergent behaviors demonstrated by the original Cellular-Potts model such as vessel sprouting, extension and anastomosis, and contraction of vascular lacunae.

**In the language of the Author Summary:**

From: Over multiple recursive evaluations, our model captures the emergent behaviors demonstrated by the original mechanistic Cellular-Potts model of as vessel sprouting, extension and anastomosis, and contraction of vascular lacunae.

To: For up to three recursive evaluations, or 300MCS, our model captures the emergent behaviors demonstrated by the original mechanistic Cellular-Potts model of as vessel sprouting, extension and anastomosis, and contraction of vascular lacunae.

Similarly, at the end of the Introduction, the authors claim:

"Our approach demonstrates the potential for deep neural networks to serve as efficient surrogate models for CPM simulations, enabling faster evaluation of computationally expensive CPM of biological processes at greater spatial and temporal scales."

Given the current model's performance, this statement is overly optimistic. While the long-term potential of such approaches is promising, this particular implementation does not yet fulfill the goals set forth in the statement.

We appreciate the reviewer's insight and make the following adjustments to our language in the Abstract and Author Summary as we did not find this language in the Introduction.

**In the language of the Abstract**, we make the following change:

From: This approach demonstrates the potential for deep learning to serve as efficient surrogate models for CPM simulations, enabling faster evaluation of computationally expensive CPM of biological processes at greater spatial and temporal scales.

To: This approach demonstrates the potential for deep learning to serve as a step toward efficient surrogate models for CPM simulations, enabling faster evaluation of computationally expensive CPM simulations of biological processes.

**In the language of the Author Summary**, we make the following change.

From: Our approach demonstrates the potential for deep neural networks to serve as efficient surrogate models for CPM simulations, enabling faster evaluation of computationally expensive CPM of biological processes at greater spatial and temporal scales.

To: Our approach demonstrates a step towards the development of surrogate models for CPM simulations using deep neural networks, enabling faster evaluation of computationally expensive CPM simulations of biological processes.

In the Conclusion, the authors write:

"Our surrogate predicts the simulation configuration significantly faster than native Cellular-Potts algorithm execution and can replicate emergent behaviors present but not explicitly defined in the Cellular-Potts model."
Again, this claim should be tempered by noting that the speed-up is only relevant for very short simulation times, beyond which the surrogate model's predictions become unreliable.

We thank the reviewer for their observations and temper our claims by adding a sentence to the conclusion.

From the conclusion: "Our surrogate predicts the simulation configuration significantly faster than native Cellular-Potts algorithm execution and can replicate emergent behaviors present but not explicitly defined in the Cellular-Potts model.

We add a sentence: "Our surrogate predicts the simulation configuration significantly faster than native Cellular-Potts algorithm execution and can replicate several multicellular emergent tissue-scale behaviors not explicitly encoded in the behaviors of individual CPM agents. While the model is effective at shorter timescales (up to 3 recurrent evaluations), its predictions diverge from the mechanistic CPM simulation over longer times."

Minor Comment

• The p-value reported in Figure 5 ($1 \times 10^{-183}$) is unnecessary. Given the visual clarity of the differences between models, such an extreme level of significance adds little value to the reader's understanding.

In response to Reviewer #2 Minor points l173, we have added more comparisons to this figure. We have taken Reviewer #1's concerns into account have made sure to be tactful in the reporting of statistics for the data in this new figure.

Reviewer #2: This paper presents a U-Net CNN as a surrogate model to predict future behaviour of a two-dimensional individual cell cellular Potts model.

1. The study seems preliminary and inconclusive. While certainly worthwhile of publication, it would be preferred if the usefulness of predicting a mere 100 MCS could be shown to be useful. For example, if the hypothesised scheme presented in the paragraph at line 234 could be shown to give quantifiable, useful results in half the time of a cellular Potts model, then the paper would have a positive story to tell.

We appreciate the reviewers concerns. However, we would like to draw the reviewer's attention to Lines 257-268 of the discussion where we discuss potential uses of the surrogate to facilitate accelerated evaluation of the Cellular-Potts model.

We also note that at a timestep of 100mcs, the vascular dynamics that are characteristic of this system can be captured and are in fact learned by our model. In fact, our system learned this effectively enough to be somewhat robust to forward propagation to forward-predict multiple timesteps, a task that the model was not trained to do.

Therefore, we raise 3 points. 1) There are exciting future directions to make use of this surrogate to accelerate the evaluation of the CPM in combination with the CPM code. 2. We have learned and documented what is easy and feasible to predict with this method and what is not, and 3) This work is useful documentation of steps towards a CPM surrogate using a neural network. Our surrogate model approach that attempts to predict the explicit model configuration is, to our knowledge, novel. Additionally, the application of a deterministic method to perform this task is otherwise unintuitive and unlikely to have succeeded as we have demonstrated (a point raised by reviewer 3). It is for these reasons that our current work is useful.

A thorough investigation into effective methods of applying this surrogate for further biological investigation is both exciting and involved. We leave this investigation for future work.

2. While it's reasonable to dismiss predicting over short/long time spans it is completely unclear why 100 MCS was selected as the quantity for prediction. Surely this is a hyper-parameter which ought to be study and tuned as part of the model?

We thank the reviewer for their concern. We would like to draw the reviewers attention to lines 75-83 in the Results section referencing Fig 1. In summary, we selected 100MCS for the following reasons: Predicting too short a timescale would result in attempting to learn stochastic noise, a seemingly infeasible task given our selected neural network architecture. At long timescales, all memory of a reference configuration is lost due in part to the stochasticity of the model. In this specific case, we observe that this memory may be lost especially because of the stochasticity of the sprouting dynamics of the vasculogenesis model. This is further discussed in reference (1), Merks et. al., 2006 where the original vasculogensis model was developed and investigated. We observed that at 100MCS, the emergent properties of vascular sprouting, branch extension and merging, and closing of small lacunae could all be observed. We also noted that these behaviors occurred consistently at the 100MCS timescale. Therefore, we expected this timescale to be a feasible problem for our deterministic U-Net model to learn. Additionally, PDE models have been

used to investigate these behaviors before thus we expect our U-Net to be able to learn these patterns.

To clarify this point, we have added the following language to Line 83:

"These three behaviors occur consistently and predictably at the 100MCS timescale and are therefore predictable enough that we may expect our model to be successful at learning these patterns. Furthermore, deterministic PDE models have previously been used to model this biological system, thus we would expect our U-Net model to be able to learn these behaviors."

In this work, we have confirmed our hypothesis that these predictable patterns may be learned by our model. We agree with the reviewer that tuning this hyperparameter may yield exciting results. Could it be possible that shorter, noisier timescales may be effectively learned by a U-Net, or could the vascular network's predictability extend to and be learned at longer timescales? We reserve this further investigation for future work.

3. The reading experience was impaired by presenting the figures in a permuted order. (The U-net architecture figure referred to as Figure 6 in the Supplementary appeared as Figure 1.)

We apologize to the reviewer and have fixed the error. We intend for this to be corrected in the resubmission.

4. Anastomosis and lacunae present different prediction problems in 3D where cellular Potts models become unfeasibly slow. Is it possible that the surrogate could be even more useful in 3D?

This is a fascinating question. Historical work with this specific vasculogenesis CPM was all performed with the 2D implementation, and this work builds upon former convolutional neural network surrogate work performed on 2D data. For these reasons and because of our concern regarding the computational expense of this investigation, we performed our work in 2D. However a 3-D implementation of the vasculogenesis model also exists for demonstration and educational purposes as a CompuCell3D demo. Extending this work to 3D using 3D convolutional neural networks and U-Nets naturally follows. Given that we've demonstrated the feasibility of our work in this paper, we would like to pursue the 3-dimenstional exploration in future work.

We have made the following addition to our discussion highlighting these points:

"Our current work currently demonstrates the feasibility of the U-Net as a CPM surrogate for 2-dimensional CPM simulations. 3D simulations using the CPM are also common but are cited as computationally expensive[1]. 3D convolutional neural networks and U-Nets have been documented as successful approaches to 3D segmentation tasks[2] and thus may be extended to serve as surrogates for 3D CPM simulations in future work."


1. Sultan S, Devi S, Mueller SN, Textor J. A parallelized cellular Potts model thatenables simulations at tissue scale; 2023. Available from: https://arxiv.org/abs/2312.09317.
2. He Y, Yu H, Liu X, Yang Z, Sun W, Anwar S, et al.. Deep Learning Based 3D Segmentation: A Survey; 2024. Available from: https://arxiv.org/abs/2103.05423

5. Ref 32 mentioned on line 32 seems to be key to whether the current study is useful or a poor substitute. What is the publication status of Ref 32?

Ref 32 is currently under review at another journal. We appreciate that the reviewer recognizes that deep generative modeling methods such as the DDPM may be better suited to learning a stochastic system. We had similar questions during the investigation of this work, and the work in that reference was inspired by this work. We fortunately reached a suitable concluding point to that exploration in time to upload it to the preprint server and cite it in this work.

Minor points
------------
l11 "of as vessel"
l235 "3 predictive steps" -> "3 predictive steps (300 MCS)" (for clarity, if that is what you mean).
l256 Hamiltonian equation appears out of nowhere. Introduce it.
l346 Fix double quotes
l173 Is it possible to show CPU/CPU or GPU/GPU time comparisons?
Ref 8: "potts" -> "Potts"
Ref 28: "Elife" -> eLife"

We have made the minor changes suggested.

Regarding l256: We unfortunately had lost a paragraph introducing the Hamiltonian when reorganizing our preprint of this work to the PLoS tex format. Thank you for pointing that out, we have added it back in.

Regarding l173: While GPU implementations of the CPM exist, and GPU implementations of the PDE-based chemical field diffusion exist, the entirety of the CPM simulation with both Cellular-Potts and diffusive-field calculations both occur on the GPU does not, to our knowledge, currently exist with an implementation of the vasculogenesis model described in this work. With this limitation in mind, we provide a neural network surrogate (GPU) to single-core CPM (CPU) comparison to demonstrate the potential speed increase that may be possible with this kind of surrogate model. However, we recognize that it may be useful to provide a comparison on equivalent hardware. Therefore, we have run further comparisons using all available threads (32) on our AMD Ryzen 5950x CPU for both the neural network surrogate and multithreaded CompuCell3D code. With this most recent evaluation, we now demonstrate a maximum speed increase by a factor of 562x comparing the surrogate on the GPU compared with single-core CPM code execution. We have updated reference to this speed increase throughout the manuscript. We have updated Figure 5 to demonstrate the additional data from this experiment and provide a data table to display the summary statistics. We have added additional language around Figure 5 and edited the figure caption as appropriate. The updated Figure 5 and new Table 1 are below:

**Table 1.** Time comparison of surrogate model evaluation compared with native CC3D code execution

| Evaluation | Median time (s) | Std. time (s) |
|---|---|---|
| Surrogate GPU | 0.004171 | 0.000877 |
| CC3D 1 core | 2.345840 | 0.203766 |
| Surrogate 32 CPU cores | 0.169648 | 0.004992 |
| CC3D 32 CPU cores | 0.607681 | 0.021977 |

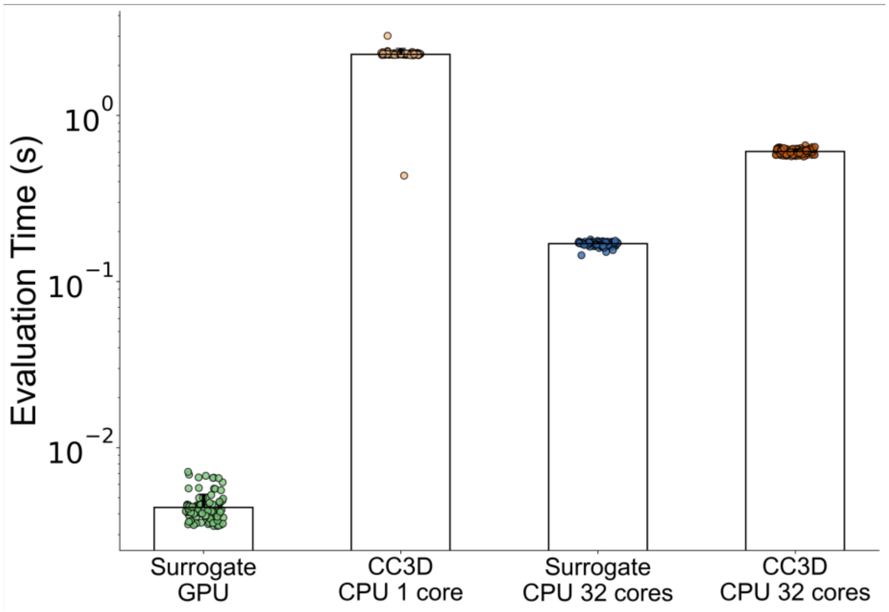


*Fig 5. Surrogate model evaluates faster than the original Cellular-Potts model in both CPU/CPU and CPU/GPU comparisons. Evaluation methods were performed for n=100 independent simulation configurations each. Median surrogate model evaluation on GPU were 0.0041s*

Reviewer #3: This paper presents an approach for surrogate modeling for the cellular Potts modeling (CPM) framework, which is evaluated on an example model of angiogenesis that involves both a diffusive field and a grid with cells.

Surrogate modeling is an interesting approach that is being applied successfully to many complex simulation methods, e.g., molecular dynamics, and so it is interesting and relevant to investigate similar speedups for the CPM. Unfortunately, I think that the results presented in this paper are still too preliminary to be broadly relevant, and I consider it unlikely that the method proposed in this paper is actually useful; it is currently more an interesting proof of concept for people working in this field, but fundamentally different methods will probably need to be used to actually do surrogate modeling for the CPM.

The main issue is simply that the CPM is a stochastic model, and so the premise of predicting the next configuration after say 100 steps is to some extent flawed. If we ran the CPM many times with different random seeds, it would also not produce the exact same configuration. However, in this

paper this task is treated as a "segmentation problem" which is fundamentally a deterministic problem. Using a deterministic surrogate for a stochastic model would have profound implications that may make it very hard or even impossible to use the surrogate for the same research questions as the original model. There may be possible ways around this; for example there could be a subclass of models where the randomness itself may not be critical and simulating a "typical" trajectory may be enough; however without any discussion of such issues or examples how this kind of surrogate could be used the paper is unfortunately too preliminary to be useful.

The authors are well aware of this issue, as they mention it in their discussion section, and have actually already begun a separate project where they use a denoising diffusion model as an architecture for the surrogate, which seems like a much better approach (https://arxiv.org/abs/2505.09630).

In summary, this manuscript would be much stronger if it showed that there is an advantage of this deterministic approach compared to the seemingly more suitable one of using a stochastic generative model as a surrogate. I cannot currently discern such an advantage from the data being shown. While the results do demonstrate that the deterministic surrogate makes predictions that retain some characteristics of the actual model, they do not demonstrate that we can still generate the same kind of insights from it.

We thank the reviewer for their stated interest in surrogate modeling and their appreciation for the problem of developing surrogates for the CPM. We would like to recognize, as we believe the reviewer has, that it may be intuitive that a stochastic generative model would be capable of serving as an effective surrogate model for the CPM. However, we also recognize that this is a hypothesis that warrants further investigation. Similarly, in this work, we investigated the hypothesis that a deterministic method like a U-Net segmentation model may be used to construct a CPM surrogate. As unlikely as this may seem, we demonstrate its success as a surrogate for the 100MCS timescale in our work. We believe that the unlikely success of a deterministic model to perform at all for learning the dynamics of a stochastic CPM is both interesting and novel work. This method may be a step towards an effective surrogate of the CPM and is therefore a useful contribution to the scientific community.

To explain its success, we refer to our response to Reviewer #2 statement 2 and the additions to the manuscript text detailed there. To summarize, the emergent properties of the CPM occur at a characteristic timescale of 100MCS and occur consistently enough at that timescale that a human observer could reasonably predict the pattern of behaviors 100MCS ahead given a model reference configuration. This suggests that those patterns are predictable even in a stochastic model. In fact, we know that the dynamics of the vascular lacunae are predictable from the prior exploration of this system using PDEs from Gamba and Serini[1-6]. Our demonstration of this is one such example of “a subclass of models where the randomness itself may not be critical and simulating a ‘typical’ trajectory may be enough” that the reviewer was searching for. A more general statement of this subclass of model would likely be any CPM model of a system that also had published attempts to study that system using PDE- based models. In vitro vessel patterning as described by the CPM model used in our study has multiple such examples of PDE models in the literature[1–6]

To both reflect this fruitful discussion in our manuscript and explicitly state a likely class of models that this method could be expected to perform well for, we add the following paragraph to the discussion:

"The CPM dynamics of vessel sprouting, branch extension and anastomosis, and lacunae contraction occur consistently enough at the 100MCS timescale that they may be considered predictable. Our work demonstrates that these consistent, predictable patterns are feasible for a deterministic U-Net architecture to learn when structured as a segmentation task, even when applied to a stochastic biological system. We would therefore expect this segmentation model approach to a CPM surrogate to yield similar success for classes of models of systems that may also be studied using other deterministic mathematical methods. For example, multiple notable PDE-based models have been developed to study in vitro vessel patterning such as the seminal work by Gamba and Serini. The investigation of this same stochastic biological process lead to the development of the CPM studied in this work."

To address the reviewers concern that "without any discussion ... or examples how this kind of surrogate could be used the paper is unfortunately too preliminary to be useful", we would like to direct the reviewers attention to the discussion paragraph beginning on line 257 Here, we describe a potential use case of a hybrid-model setup whereby the surrogate model could be used in an alternating fashion alongside the mechanistic CPM to both accelerate computation and re-set the reference configuration using the CPM to mitigate the inherent error introduced by the convolution and pooling operations of the U-Net.

To address the reviewer's concern that "this manuscript would be much stronger if it showed that there is an advantage of this deterministic approach compared to the seemingly more suitable one of using a stochastic generative model as a surrogate", we add the following statement to the discussion:

"However, our denoising diffusion models demonstrate speed increases that are far less dramatic than those demonstrated by the model described in this work."

The denoising diffusion probabilistic model (DDPM) as described in that work is established as an effective, even state-of-the-art, generative modeling method. Those models involve training a neural network, often a U-Net, to progressively remove noise from input images over multiple evaluations.  This reveals a generated output from the same statistical distribution of the learned training data. This would intuitively result in much slower evaluation as generating a sample requires hundreds to thousands of iterative denoising steps. Each denoising step is a forward pass of data through the trained U-Net. This would likely take more time and computational resources compared with the single forward pass required to predict 100MCS from our model, thus explaining the less competitive speed increase. We therefore expect that the DDPM may be effective for sampling a statistically equivalent model configuration at long time horizons of 20,000MCS as opposed to predicting forward 100MCS. The investigation of that expectation is what is documented in that preprint the reviewer referenced.

Many other generative modeling methods exist, however a thorough review of those methods and an attempt to apply and compare their fitness to be a surrogate for the Cellular-Potts method is very involved and best left for future work.

To address the reviewer's concern of "While the results do demonstrate that the deterministic surrogate makes predictions that retain some characteristics of the actual model, they do not demonstrate that we can still generate the same kind of insights from it". We understand why it would be nice to have a perfect replicate of the mechanistic model and we agree that we're not there yet. We do feel that it is important to discuss what is and is not possible with this method (as we have discussed above with Reviewer 2 and in the manuscript). This work represents a significant step towards our and the reviewer's shared vision of a perfect replica of the CPM that simulates significantly faster.

I would now like to raise a point for discussion to address the concern Reviewer 2 and 3 both may have regarding suitability of this work for direct application as-is for investigating biological processes. This work documents the development of a model surrogate which approaches but may not be a 1-to-1 replica of the underlying numerical method. This particular model surrogate is a neural network, or a *neural operator*, that may be used to augment the underlying numerical method (in our case, the Cellular-Potts method), for accelerating the simulation. Classic numerical methods alone for simulation has been cited as intractable for large-scale "real-world" problems in fields such as weather forecasting, material science, fluid dynamics among others. These points are discussed in the following review: https://www.nature.com/articles/s42254-024-00712-5. While our work focuses very much on the development of a surrogate, we believe that practitioners of computational biology, many of whom use the CPM in their investigations, deserve to benefit from the investigation of surrogate modeling techniques as the other cited computational fields have. We also believed that the computational biology audience would find this work interesting as all three reviewers have stated. It is for this reason that we have chosen to send our manuscript to PLoS Computational Biology.


1. Serini, G., Ambrosi, D., Giraudo, E., Gamba, A., Preziosi, L., and Bussolino, F. (2003). Modeling the early stages of vascular network assembly. EMBO J. *22*, 1771–1779. 10.1093/emboj/cdg176.

2. Namy, P., Ohayon, J., and Tracqui, P. (2004). Critical conditions for pattern formation and in vitro tubulogenesis driven by cellular traction fields. J. Theor. Biol. *227*, 103–120. 10.1016/j.jtbi.2003.10.015.

3. Murray, J.D. (2003). On the mechanochemical theory of biological pattern formation with application to vasculogenesis. C. R. Biol. *326*, 239–252.

4. Manoussaki, D., Lubkin, S.R., Vernon, R.B., and Murray, J.D. (1996). A mechanical model for the formation of vascular networks in vitro. Acta Biotheor. *44*, 271–282. 10.1007/BF00046533.

5. Gamba, A., Ambrosi, D., Coniglio, A., de Candia, A., Di Talia, S., Giraudo, E., Serini, G., Preziosi, L., and Bussolino, F. (2003). Percolation, morphogenesis, and burgers dynamics in blood vessels formation. Phys. Rev. Lett. *90*, 118101. 10.1103/PhysRevLett.90.118101.

6. Ambrosi, D., Gamba, A., and Serini, G. (2004). Cell directional persistence [corrected] and chemotaxis in vascular morphogenesis. Bull. Math. Biol. *66*, 1851–1873. 10.1016/j.bulm.2004.04.004.

---

**Have the authors made all data and (if applicable) computational code underlying the findings in their manuscript fully available?**

The PLOS Data policy requires authors to make all data and code underlying the findings described in their manuscript fully available without restriction, with rare exception (please refer to the Data Availability Statement in the manuscript PDF file). The data and code should be provided as part of the manuscript or its supporting information, or deposited to a public repository. For example, in addition to summary statistics, the data points behind means, medians and variance measures should be available. If there are restrictions on publicly sharing data or code —e.g. participant privacy or use of data from a third party—those must be specified.

Reviewer #1: Yes

Reviewer #2: Yes

Reviewer #3: Yes

---

PLOS authors have the option to publish the peer review history of their article (what does this mean?). If published, this will include your full peer review and any attached files.

If you choose “no”, your identity will remain anonymous but your review may still be made public.

**Do you want your identity to be public for this peer review?** For information about this choice, including consent withdrawal, please see our Privacy Policy.

Reviewer #1: No

Reviewer #2: No

Reviewer #3: No

**Figure resubmission:**

While revising your submission, please upload your figure files to the Preflight Analysis and Conversion Engine (PACE) digital diagnostic tool, https://pacev2.apexcovantage.com/. PACE helps ensure that figures meet PLOS requirements. To use PACE, you must first register as a user.

Registration is free. Then, login and navigate to the UPLOAD tab, where you will find detailed instructions on how to use the tool. If you encounter any issues or have any questions when using PACE, please email PLOS at figures@plos.org. Please note that Supporting Information files do not need this step. If there are other versions of figure files still present in your submission file inventory at resubmission, please replace them with the PACE-processed versions.

**Reproducibility:**

To enhance the reproducibility of your results, we recommend that authors of applicable studies deposit laboratory protocols in protocols.io, where a protocol can be assigned its own identifier (DOI) such that it can be cited independently in the future. Additionally, PLOS ONE offers an option to publish peer-reviewed clinical study protocols. Read more information on sharing protocols at https://plos.org/protocols?utm_medium=editorial-email&utm_source=authorletters&utm_campaign=protocols
